# Crystal structures of three cyclohexane-based γ-spirolactams: determination of configurations and conformations

**DOI:** 10.1186/s13065-019-0586-7

**Published:** 2019-05-17

**Authors:** Tobias Krueger, Alexandra Kelling, Torsten Linker, Uwe Schilde

**Affiliations:** Institute of Chemistry, University of Potsdam, Karl-Liebknecht-Str. 24-25, 14476 Potsdam, Germany

**Keywords:** 2-Azaspiro[4.5]deca-1-ones, *Cis*- and *trans*-form, Configuration, Conformation, Lactams

## Abstract

The title compounds, 2-azaspiro[4.5]deca-1-one, C_9_H_15_NO, (**1a**), *cis*-8-methyl-2-azaspiro[4.5]deca-1-one, C_10_H_17_NO, (**1b**), and *trans*-8-methyl-2-azaspiro[4.5]deca-1-one, C_10_H_17_NO, (**1c**), were synthesized from benzoic acids **2** in only 3 steps in high yields. Crystallization from *n*-hexane afforded single crystals, suitable for X-ray diffraction. Thus, the configurations, conformations, and interesting crystal packing effects have been determined unequivocally. The bicyclic skeleton consists of a lactam ring, attached by a spiro junction to a cyclohexane ring. The lactam ring adopts an envelope conformation and the cyclohexane ring has a chair conformation. The main difference between compound **1b** and compound **1c** is the position of the carbonyl group on the 2-pyrrolidine ring with respect to the methyl group on the 8-position of the cyclohexane ring, which is *cis* (**1b**) or *trans* (**1c**). A remarkable feature of all three compounds is the existence of a mirror plane within the molecule. Given that all compounds crystallize in centrosymmetric space groups, the packing always contains interesting enantiomer-like pairs. Finally, the structures are stabilized by intermolecular N–H···O hydrogen bonds.

**Electronic supplementary material:**

The online version of this article (10.1186/s13065-019-0586-7) contains supplementary material, which is available to authorized users.

## Introduction

**Fig. 1:**
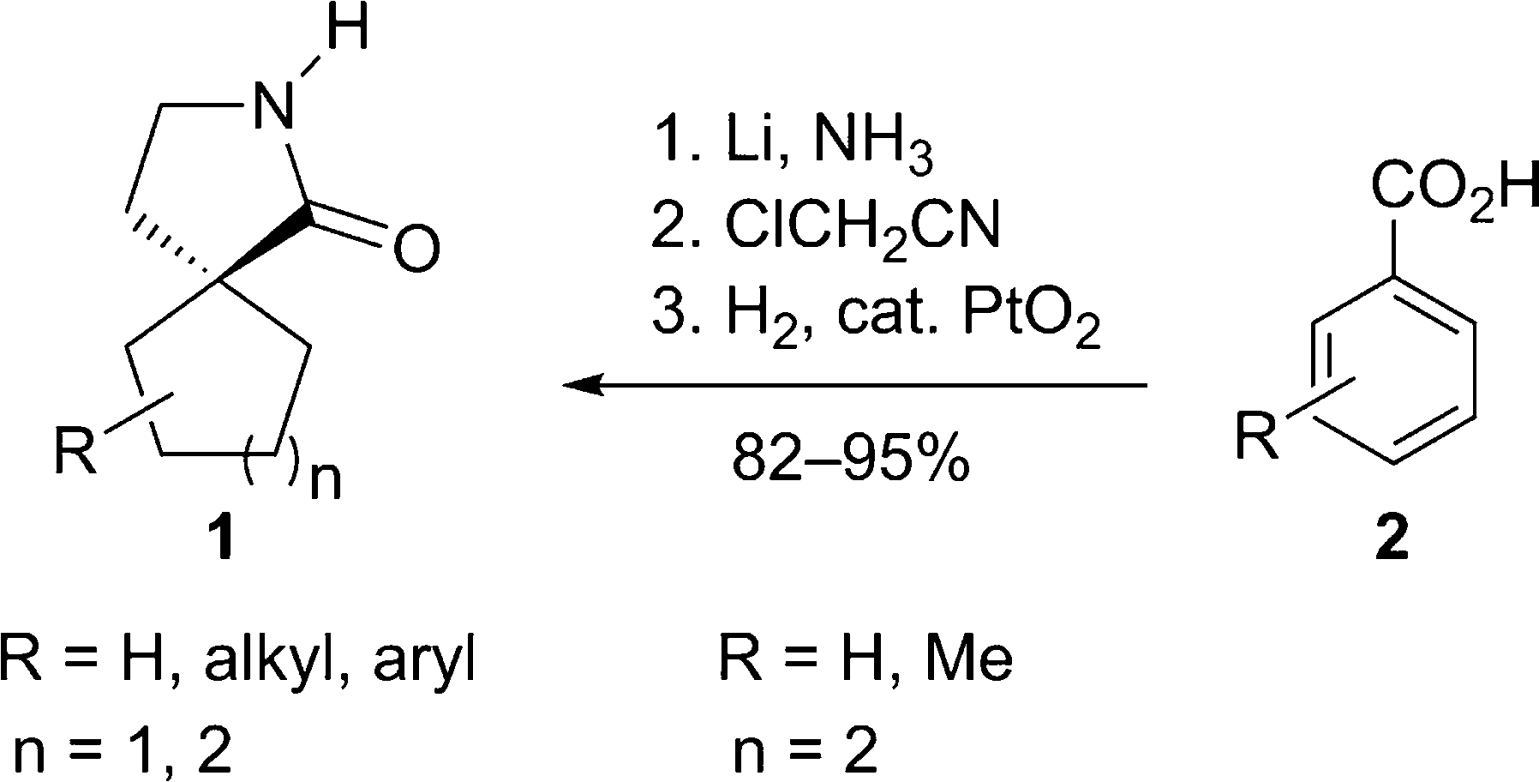
Examples of γ-spirolactams **1** and their synthesis from benzoic acids **2**

γ-Spirolactams of the general structure **1** (Fig. 1) are interesting organic heterocycles and have attracted much attention as potential medicinal agents [1]. They can be synthesized by various methods, but over many steps [2–4]. Cyclohexane-based γ-spirolactams **1** (n = 2) have been described to bind to opioid receptors and as enzyme inhibitors in two patents [5, 6]. Very recently, we developed an easy entry to such compounds by Birch reduction of the corresponding benzoic acids **2** (R = H, Me) and subsequent alkylation and hydrogenation (Fig. 1) [7].

**Fig. 2:**
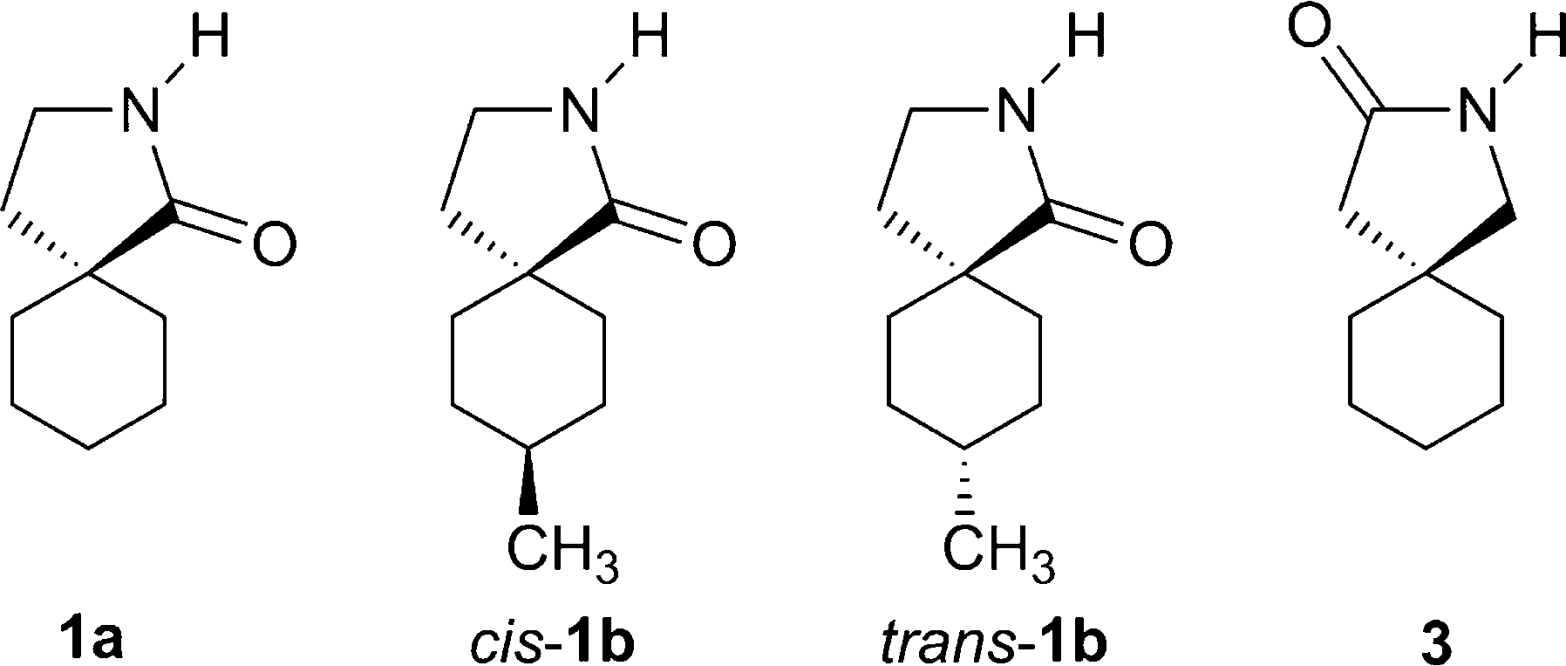
Herein investigated γ-spirolactams **1a**, *cis*-**1b** and *trans*-**1b** and known Gabapentin lactam **3**

During the synthesis of γ-spirolactams **1**, we obtained different regio- and stereoisomers, some configurations have been already established in our previous studies [7]. However, in the 8-methyl derivatives **1b** *cis* and *trans* isomers have been isolated, with functional groups too far away from each other to apply NOE NMR experiments for structure elucidation. Therefore, we had to obtain single crystals to determine the structures unequivocally by X-ray diffraction. Besides the configurations, the conformations of γ-spirolactams **1** are interesting, since the six-membered ring will adopt a chair form, which might influence the more flexible five-membered lactam ring. Indeed, crystal structures of simple γ-butyrolactams indicate that the substitution by an additional methyl group influences the conformations remarkably [8–10]. Herein, we report three new crystal structures of cyclohexane-based γ-spirolactams **1a**, *cis*-**1b** and *trans*-**1b**. We could determine their configurations and conformations, which will be discussed in detail. Our results should be interesting in comparison with the similar Gabapentin lactam **3** (Fig. 2), whose conformation has already been analyzed in the solid state [11] and which has been found to be neuroprotective in retinal ischemia [12].

## Results and discussion

**Fig. 3:**
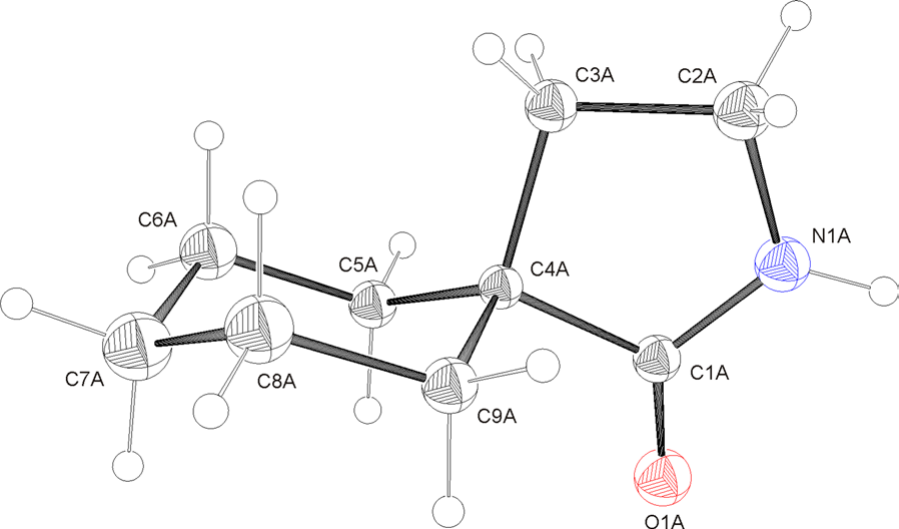
Molecular structure of **1a**, showing the atom labeling and 30% probability ellipsoids. H atoms as small circles of arbitrary radius. The asymmetric unit contains four molecules (*A*–*D*), only one molecule is drawn

γ-Spirolactams **1** have been synthesized from the corresponding benzoic acids **2** in only few steps in high yields (Fig. 1, Experimental section). Single crystals have been obtained by crystallization from *n*-hexane. We started our investigations with the unsubstituted lactam **1a**, which crystallizes in the monoclinic space group *C*2/*c*, with 32 molecules per unit cell (Fig. 3).

The lactam ring adopts an envelope conformation, whereas the cyclohexane ring has an almost ideal chair conformation. There are four molecules in the asymmetric unit of γ-spirolactam **1a**, which show small conformational differences. The bond lengths within the cyclohexane ring lie in the expected ranges [1.514(3)–1.549(2) Å], whereas one bond to the spiro atom C4 is somewhat longer than the other ones. The bond angles range from 109.56(13)° to 112.48(15)°. The smallest angle occurs at the spiro junction. However, the bond angle is the biggest one on that carbon atom, which is adjacent to the spiro atom and bound to that with the longest bond. The conformations of the cyclohexane rings only slightly deviate from the ideal chair [*θ* = 177.9(2)°/180.00(19)°/178.09(19)°/178.6(2)° for molecules *A*–*D*, respectively]. The puckering amplitudes *q* are 0.566(2), 0.5691(19), 0.564(2) and 0.568(2) Å. The displacement parameters *φ* are 313(7)°, 293(10)°, 66(8)° and 309(8)°. The torsion angles slightly deviate (maximal deviation: 2.46°) from the value observed in cyclohexane (55.9°). The dihedral angles between the cyclohexane rings and the pyrrolidine rings are 79.931(68)°, 80.112(68)°, 79.975(66)° and 80.034(70)°, whereby the spiro atom C4 has a maximum deviation from the mean plane of the five-membered ring [*A*: 0.0331(17), *B*: 0.0276(13), *C*: − 0.0301(17), *D*: − 0.0342(14) Å]. The twisting of both rings to each other can also be expressed by the torsion angles including the spiro atom C4 (for details see Additional file 1).

Importantly, the carbonyl group of lactam **1a** occupies the *pseudo* equatorial position in the cyclohexane ring, because it is sterically more demanding than the methylene group of the lactam ring, which is axial. The preference of bulky substituents in the equatorial position of cyclohexanes is well-known [13, 14]. Furthermore, our X-ray structure is in accordance to Gabapentin lactam **3** in the solid state [11], which is a regioisomer of γ-spirolactam **1a** (Fig. 2), showing the sterically more demanding carbonyl group in the *pseudo* equatorial position as well.

The lactam rings adopt a slightly distorted envelope conformation, characterized by the puckering parameters *q* = 0.2800(19)°/0.2723(19)°/0.2820(19)°/0.2787(19)° Å and *φ* = 104.6(4)°/104.6(4)°/284.2(4)°/104.7(4)° for molecules *A*–*D* (theoretical values for envelope: 108/288°). The torsion angles around the ring bonds deviate up to 27.18(18)° (C1C–C4C–C3C–C2C) from 0° of a perfectly planar ring system. As expected, around the amide bond N1–C1 is the smallest torsion angle of − 1.3(2)° (C2A–N1A–N1A–C4A). The C–C–C bond angles within the five-membered rings are 101.84(13)°–108.84(14)° (*A*), 102.19(13)°–108.38(16)° (*B*), 101.75(13)°–108.73(14)° (*C*), and 102.22(13)°–108.41(16)° (*D*). The smallest values occur including the spiro atom, the biggest at the carbonyl group. The bond angles including the nitrogen atom range from 114.15(14)° (*A*) to 114.61(15)° (*B*). The amide (N1–C1) bond lengths from 1.330(2) to 1.336(2) Å indicate partial double-bond character.

**Fig. 4:**
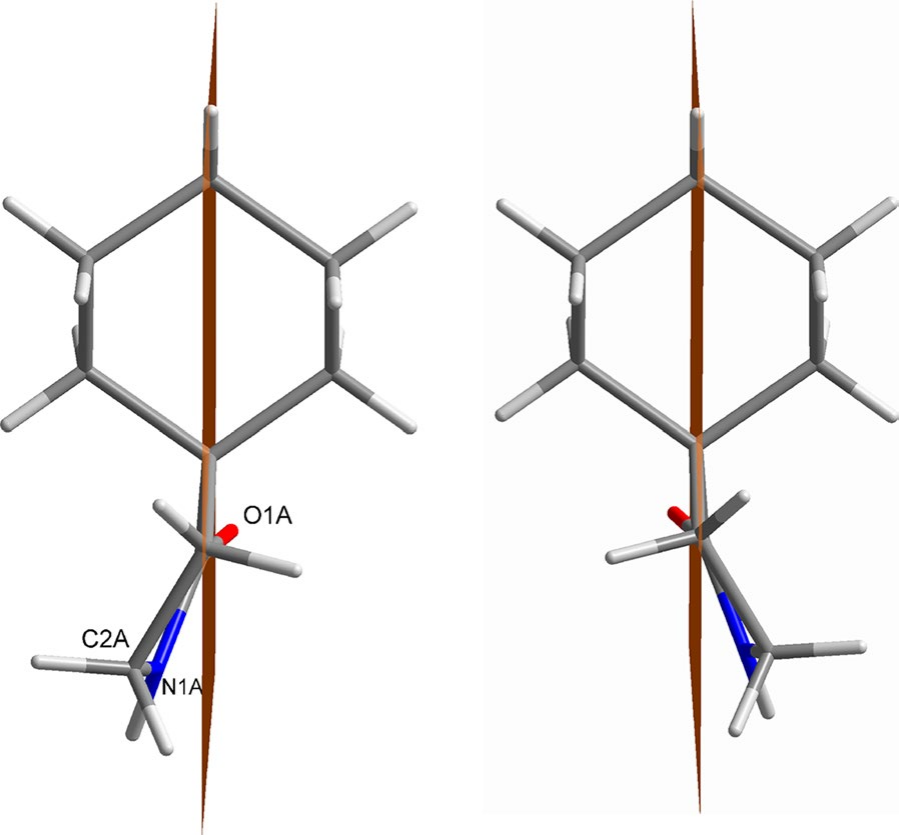
Mirror plane (brown) and the orientation of the atoms O1, N1, and C2 (molecule A) with respect to this plane in two conformers of γ-spirolactam **1a**

Interestingly, a mirror plane can be put through the atoms C4 and C7, resulting in two mirror images (Fig. 4), with exactly opposite orientation of the lactam rings like in enantiomers. Such a behavior can be described as dynamic stereoisomerism [15] and is known from X-ray structures of simple γ-butyrolactam as well [10]. Therefore, although the lactam ring is in solution very flexible and can adopt different envelope conformations, it is fixed in the solid state by hydrogen bonds.

**Fig. 5:**
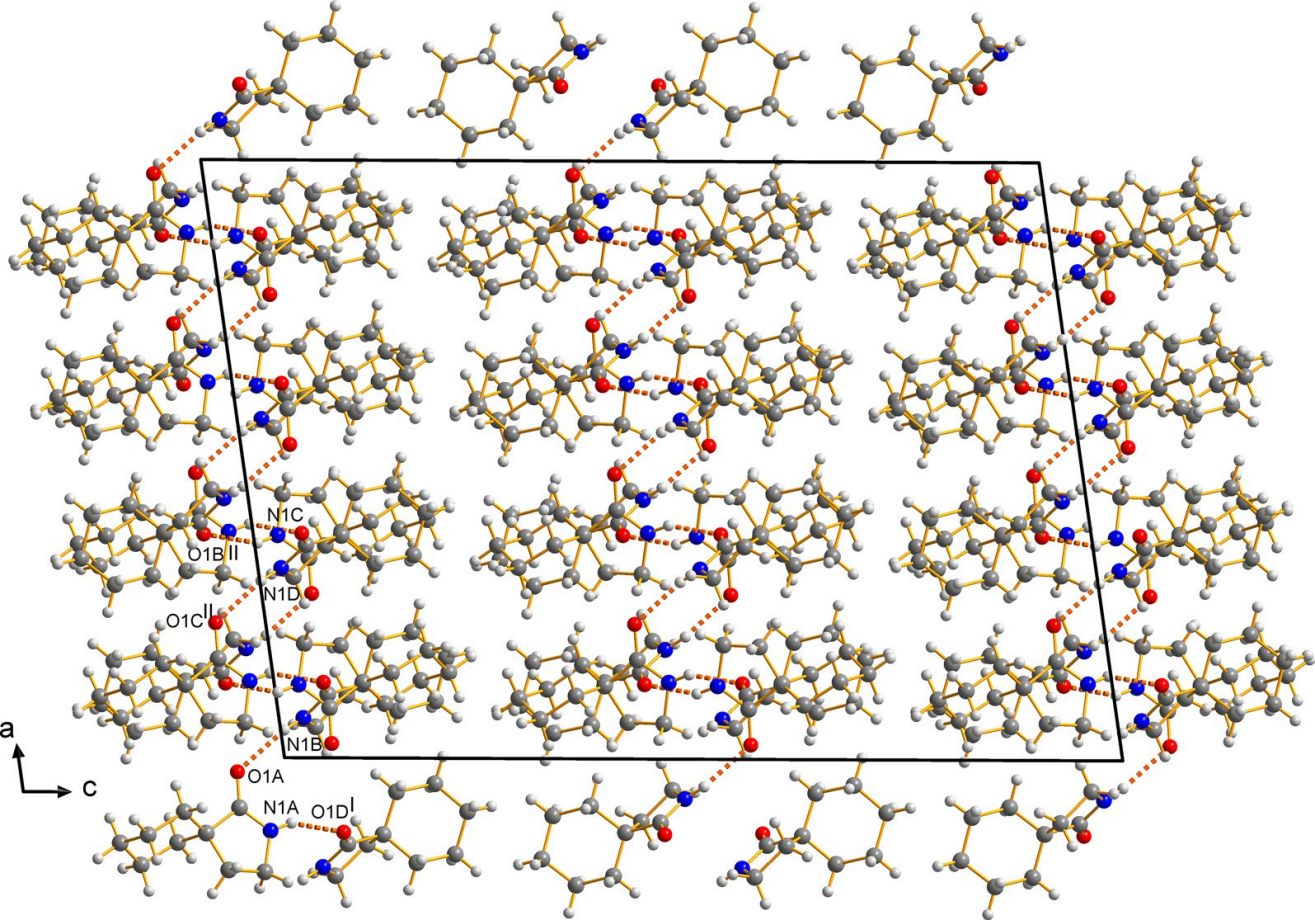
Crystal packing of **1a**, illustrating the hydrogen bonds (dashed lines). View along *b*

The maximal deviations from that plane are 0.0276(13) to − 0.0342(14) Å (C4). Due to the opposite positions of the other lactam ring atoms (O1, N1, C2), with respect to that plane, pairs always occur. In accordance with the envelope conformation, the distances to the plane are significant [O1: 0.2927(27) (*B*) to 0.2988(32) Å (*A*), N1: − 0.4394(21) (*B*) to − 0.4651(21) Å (*D*), C2: − 0.7110(24) (*B*) to − 0.7412(24) Å (*D*)]. The packing is stabilized by N–H···O hydrogen bonds, which are located approximately along the crystallographic *c* axis as well as in between *a* and *c* forming a 2*D*-network with double strands in the *a* direction (Fig. 5; for details of the hydrogen-bonding geometry, see Table 1).

**Table 1:** Hydrogen bond geometries (Å, °) for **1a**, *cis*-**1b** and *trans*-**1b**

	D–H	H···A	D···A	D–H···A	
**1a**					
N1A–H1A···O1D	0.88(2)	2.10(3)	2.913(2)	154(2)	x − 0.5, y − 0.5, z
N1B–H1B···O1A	0.85(2)	2.13(2)	2.917(2)	154(2)	–
N1C–H1C···O1B	0.86(2)	2.14(2)	2.940(2)	154(2)	0.5 − *x*, 1.5 − *y*, − *z*
N1D–H1D···O1C	0.89(2)	2.10(2)	2.934(2)	155(2)	0.5 − *x*, 1.5 − *y*, − *z*
*Cis*-**1b**					
N1–H1···O1	0.87(2)	2.00(2)	2.872(1)	174(1)	1 − x, 1 − y, 1 − z
*Trans*-**1b**					
N1–H1···O1	0.88(1)	1.991(1)	2.835(1)	162(1)	*x*, 0.5 − *y*, 0.5 + *z*

**Fig. 6:**
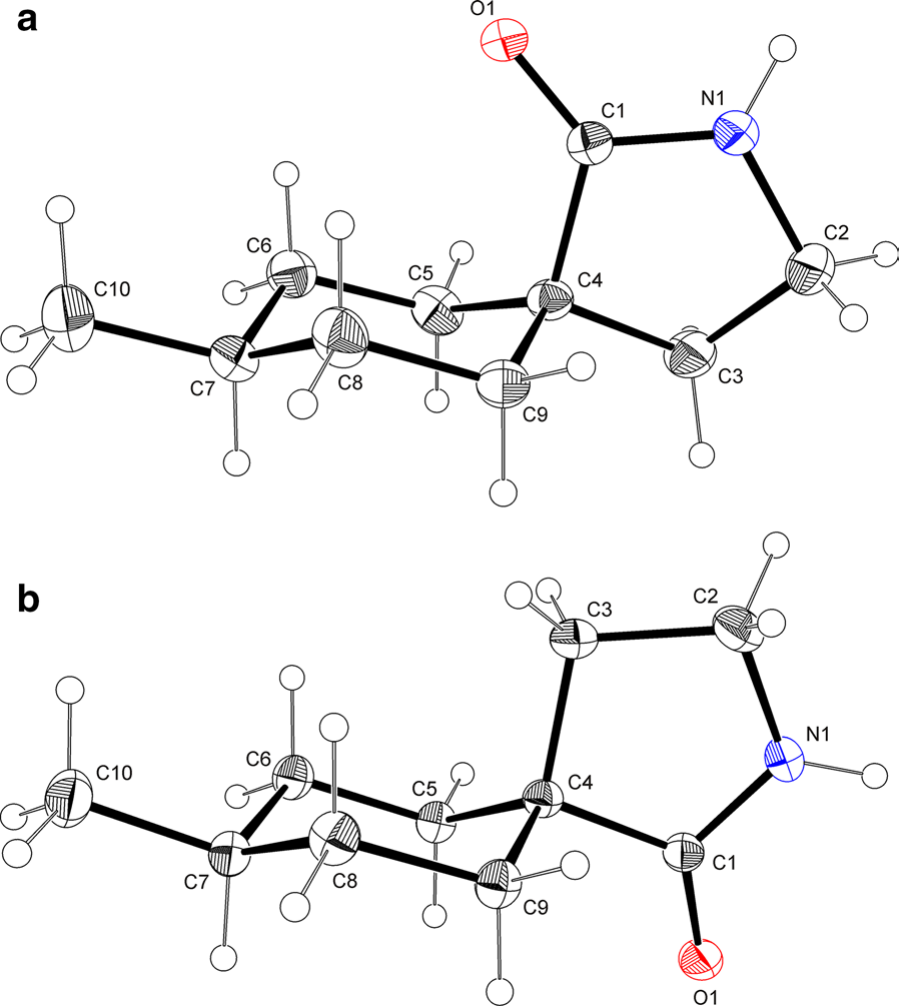
Molecular structures of *cis*-**1b** (**a**) and *trans*-**1b** (**b**), showing the atomic labeling and 30% probability ellipsoids. H atoms as small circles of arbitrary radius

We investigated γ-spirolactams **1b** next, which crystallize in the monoclinic space group *P*2_1_/*c*, with one molecule per asymmetric unit. Indeed, it was now possible to determine the relative configurations and assign *cis* and *trans* isomers unequivocally (Fig. 6). Similar to derivative **1a**, a mirror plane can be found between atoms C4, C10, H7, and H10, with the lactam ring slightly out of this plane in different envelope conformations. Interestingly, in both isomers the methyl group occupies the equatorial position in the cyclohexane ring, due to its steric demand [13, 14]. The conformation of the lactam is more flexible and therefore controlled by this methyl group. Thus, the carbonyl group is forced *pseudo* axial in *cis*-**1b** and *pseudo* equatorial in *trans*-**1b** (Fig. 6).

**Fig. 7:**
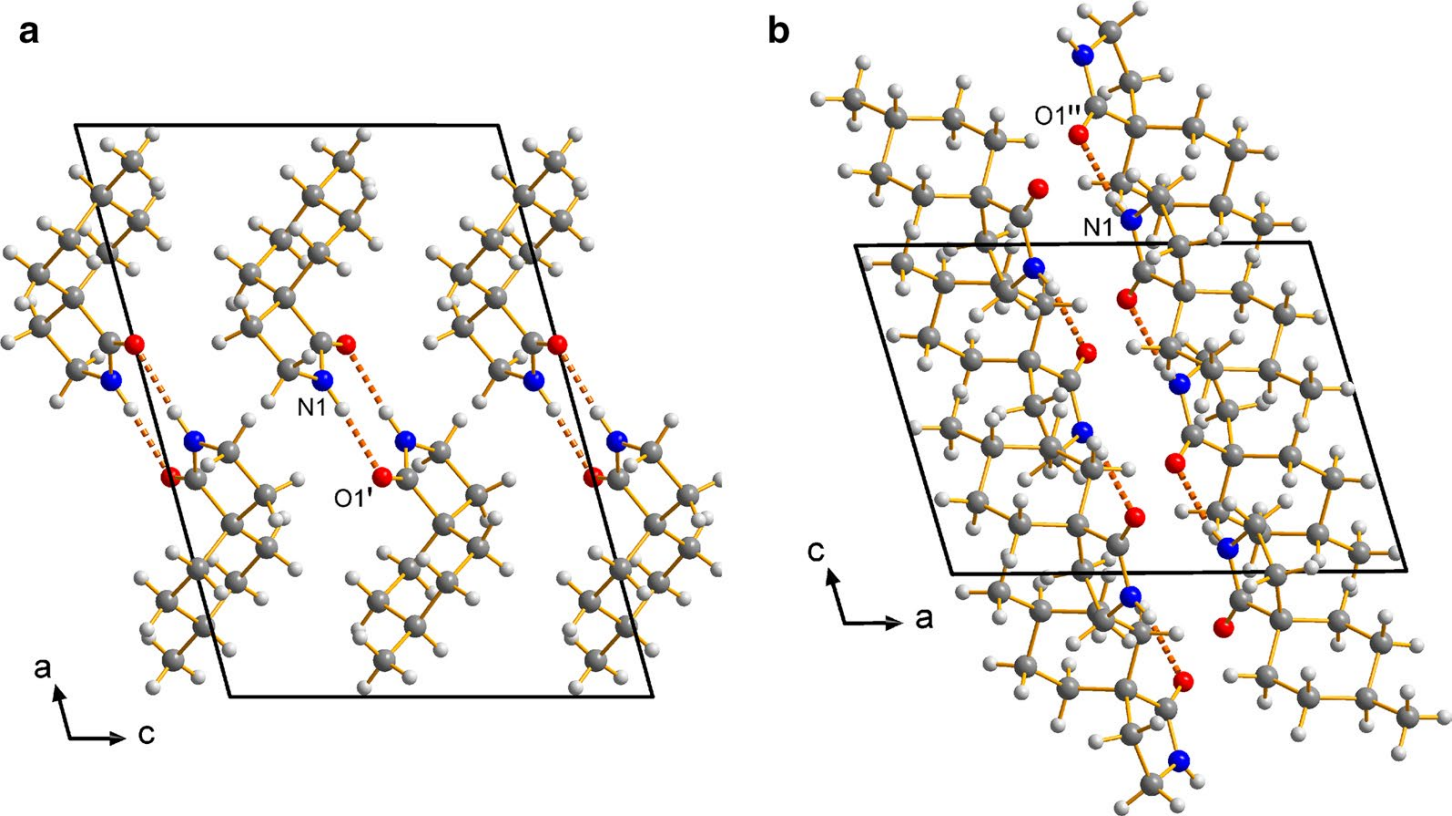
Crystal packing of *cis*-**1b** (**a**) and *trans*-**1b** (**b**), illustrating the hydrogen bonds (dashed lines)

The crystal packings of the two lactams **1b** are different (Fig. 7). The molecules of *cis*-**1b** crystallize as centrosymmetric dimers, where the molecules are held together by two N–H···O hydrogen bonds running along the crystallographic *a* axis. The central unit of *trans*-**1b** forms 1*D*-chains along the crystallographic *c* axis, fixed by hydrogen bonds (Table 1, Fig. 7). In comparison with **1a**, we can find stronger hydrogen bonds in *cis*-**1b** and *trans*-**1b**, in conclusion of the shorter D···A distances and the D–H···A angles, which are closer to 180°. These findings are in accordance with the N–H stretching frequencies in IR spectra—usually observed from 3500 to 3200 cm^−1^ for non-associated N–H–but here shifted to lower frequencies due to hydrogen bonds (**1a**: 3292 cm^−1^), but even lower in *cis*-**1b** and *trans*-**1b** (3201 and 3211 cm^−1^).

The structural features of **1b** are closely related to those in **1a**. Table 2 lists the geometric parameters in detail. The biggest deviation from the ideal chair conformation of the cyclohexane ring can be observed in *cis*-**1b**.

**Table 2:** Geometrical parameters for γ-spirolactams **1b** (Å, °)

	*cis*-**1b**	*trans*-**1b**
6-membered ring		
Bond lengths	1.5188(19)–1.5413(15)	1.5237(15)–1.5465(14)
Bond angles	109.74(10)–114.24(10)	109.08(8)–112.71(9)
Puckering: *q*/*θ*/*φ*	0.5500(14)/174.57(15)/29.8(2)	0.5676(12)/176.49(12)/208(2)
Torsion angles	49.00(14)–57.57(14)	52.52(12)–57.52(12)
5-membered ring		
C1–N1	1.3314(15)	1.3316(14)
C–C–C	100.79(9)–108.71(10)	102.30(8)–109.09(9)
C1–N1–C2	114.37(10)	114.1(1)
Puckering: *q*/*φ*	0.2995(14)/282.7(2)	0.2546(12)/108.2(3)
Torsion angles	2.56(13)–29.11(12)	0.75(13)–24.63(12)
6- to 5-memb. ring		
Dihedric angle	81.806(43)	81.007(42)
Torsion angles		
C1–C4–C5–C6	− 73.02(13)	− 177.14(8)
C1–C4–C9–C8	73.72(13)	− 176.63(8)
C3–C4–C9–C8	− 176.41(10)	70.74(11)
C3–C4–C5–C6	173.04(10)	− 68.60(1)
Lactam ring plane		
Max. deviation (C4)	− 0.0299(10)	0.0208(9)
Distance to O1	− 0.3384(19)	− 0.2321(18)
Distance to N1	0.4900(16)	0.3764(14)
Distance to C2	0.7795(19)	0.6460(19)

## Conclusions

In conclusion, we could easily synthesize three different γ-spirolactams from benzoic acids. Their configurations could not be determined by NMR spectroscopy, and thus X-ray analysis was the method of choice. We could unequivocally establish *cis*- and *trans*-isomers for the first time. Despite the configurations, we could additionally determine the conformations of the γ-spirolactams in the solid state. Thus, the cyclohexane rings adopt almost ideal chair forms, although the spiro junction leads to some distortion. Importantly, the steric demand of the methyl groups, which are always oriented pseudo equatorial, controls the conformations. Finally, we found an interesting behavior in the crystal packing, controlled by intermolecular hydrogen bonds between the acidic NH protons and the basic C=O groups. Thus, mirror planes were found *between* two crystallized molecules, affording enantiomer-like pairs. Since the herein investigated γ-spirolactams are very similar to Gabapentin lactam, which is neuroprotective in retinal ischemia, our studies might be interesting for biological or medical applications.

## Experimental section

### General synthetic

Melting points (m.p.) were determined by using a Mel-Temp from Electrothermal. TLC was performed using TLC Silica gel 60 F254 aluminium sheets from Merck. ^1^H NMR and ^13^C NMR spectra were measured by using a Bruker Avance 500 (500 MHz, 125 MHz) or a Bruker Avance 600 (600 MHz, 150 MHz) NMR spectrometer. Signals were assigned by two-dimensional methods (COSY, HMQC, HMBC). The IR spectra were recorded in KBr pellets by using a Nicolet 6700 FT-IR spectrometer from Thermo Electron Corporation. Elemental analysis was performed on a Vario EL III elemental analyzer. All starting materials were used as purchased without further purification.

### γ-Spirolactams **1a**, *cis*-**1b**, and *trans*-**1b**

Benzoic acid (**2a**) (12.21 g, 100 mmol) or p-toluic acid (13.62 g, 100 mmol) were introduced into a three-necked flask (500 mL), equipped with a dry-ice condenser and cooled to − 78 °C by a dry-ice acetone bath. Ammonia (200 mL) was condensed into the three-necked flask, and lithium (1.39 g, 200 mmol) was added in small pieces to the solution at − 78 °C, until it remained blue. After stirring for 1 h at − 78 °C, chloroacetonitrile (10.0 mL, 160 mmol) was added via syringe within 2 min and the ammonia was allowed to evaporate overnight at RT. The solid residue was dissolved in water (70 mL), cooled to 0 °C, acidified with 6 M HCl to pH 2 and extracted with dichloromethane (3 × 50 mL). The combined organic layers were dried over sodium sulfate, filtered over a small pad of silica gel, and the solvent was removed in vacuo. The crude products were crystallized from n-hexane to obtain the intermediate cyclohexadienes **4a** (15.7 g, 96%) or **4b** (16.3 g, 92%) as white solids. Cyclohexadiene **4b** was obtained as a 55:45 mixture of diastereomers, which were separated by column chromatography (silica gel, hexane/ethyl acetate/methanol 3:1:0.25) to afford 4.9 g (28%) of *cis*-**4b**, 5.2 g (29%) of *trans*-**4b** and 5.9 g (33%) of a mixture fraction. This fraction was separated again under same conditions to afford additionally 2.5 g (14%) of *cis*-**4b** and 3.0 g (17%) of *trans*-**4b** in analytically pure forms.
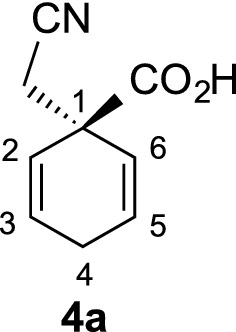


#### Cyclohexadiene **4a**

*R*_f_ = 0.55 (CH_2_Cl_2_/MeOH/HOAc 9:1:0.1); m.p. 122–123 °C; ^1^H NMR (500 MHz, CDCl_3_): *δ* 10.08 (brs, 1 H, O–H), 6.13 (dt, *J *= 10.3, 3.3 Hz, 2 H, 3-H, 5-H), 5.75 (dt, *J *= 10.3, 1.9 Hz, 2 H, 2-H, 6-H), 2.72–2.79 (m, 2 H, 4-H), 2.74 (s, 2 H, CH_2_CN); ^13^C NMR (125 MHz, CDCl_3_): *δ* 26.2 (t, CH_2_CN), 28.4 (t, C-4), 46.0 (s, C-1), 116.5 (s, CN), 123.5 (d, C-2, C-6), 129.3 (d, C-3, C-5), 177.7 (s, CO_2_H); IR (KBr) $\widetilde{v}$ = 3164, 2965, 2270, 1724, 1227, 754 cm^−1^; elemental analysis calcd (%) for C_9_H_9_NO_2_ (163.18): C 66.25, H 5.56, N 8.58; found: C 65.93, H 5.66, N 8.56.
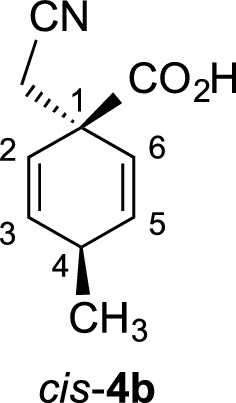


#### Cyclohexadiene *cis*-**4b**

*R*_f_ = 0.58 (CH_2_Cl_2_/MeOH/HOAc 9:1:0.1); m.p. 143–144 °C; ^1^H NMR (500 MHz, CDCl_3_): *δ* 10.32 (brs, 1 H, O–H), 6.04 (dd, *J *= 10.3, 3.4, Hz, 2 H, 3-H, 5-H), 5.69 (dd, *J *= 10.3, 2.0 Hz, 2 H, 2-H, 6-H), 2.83–2.89 (m, 1 H, 4-H), 2.73 (s, 2 H, CH_2_CN), 1.14 (d, *J *= 7.4 Hz, 3H, CH_3_); ^13^C NMR (125 MHz, CDCl_3_): *δ* 21.0 (q, CH_3_), 28.2 (t, CH_2_CN), 30.7 (t, C-4), 46.1 (s, C-1), 116.6 (s, CN), 122.3 (d, C-2, C-6), 135.6 (d, C-3, C-5), 177.8 (s, CO_2_H); IR (KBr) $\widetilde{v}$ = 3140, 2939, 2272, 1726, 1227, 755 cm^−1^; elemental analysis calcd (%) for C_10_H_11_NO_2_ (177.20): C 67.78, H 6.28, N 7.90; found: C 67.74, H 6.06, N 7.90.
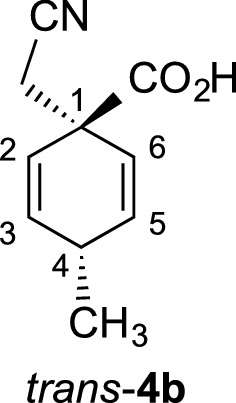


#### Cyclohexadiene *trans*-**4b**

*R*_f_ = 0.52 (CH_2_Cl_2_/MeOH/HOAc 9:1:0.1); m.p. 161–162 °C; ^1^H NMR (500 MHz, CDCl_3_): *δ* 11.42 (brs, 1 H, O–H), 6.06 (dd, *J *= 10.3, 3.3, Hz, 2 H, 3-H, 5-H), 5.70 (dd, *J *= 10.3, 2.0 Hz, 2 H, 2-H, 6-H), 2.85–2.92 (m, 1 H, 4-H), 2.78 (s, 2 H, CH_2_CN), 1.22 (d, *J *= 7.4 Hz, 3H, CH_3_); ^13^C NMR (125 MHz, CDCl_3_): *δ* 21.4 (q, CH_3_), 28.5 (t, CH_2_CN), 30.8 (t, C-4), 46.3 (s, C-1), 116.7 (s, CN), 122.4 (d, C-2, C-6), 135.6 (d, C-3, C-5), 176.6 (s, CO_2_H); IR (KBr) $\widetilde{v}$ = 3164, 2965, 2270, 1725, 1227, 754 cm^−1^; elemental analysis calcd (%) for C_10_H_11_NO_2_ (177.20): C 67.78, H 6.28, N 7.90; found: C 67.66, H 6.21, N 7.94.

The cyclohexadienes **4a** (3.26 g, 20 mmol), *cis*-**4b** (3.54 g, 20 mmol), or *trans*-**4b** (3.54 g, 20 mmol) were dissolved in methanol (300 mL) at RT and platinum(iv) oxide (50 mg, 1 mol%) and 37% HCl (1.5 mL) was added. The solution was purged with hydrogen gas for 5 min, equipped with a balloon filled with hydrogen gas and hydrogenated under stirring for 48 h. The solution was filtered through a pad of Celite, washed with methanol (2 × 50 mL) and the solvent was removed in vacuo. The residue was dissolved in pyridine (500 mL) and heated for 8 h under reflux. The pyridine was removed in vacuo and the residue was dissolved in dichloromethane (200 mL), extracted with 1 N HCl (100 mL), dried over sodium sulfate, and concentrated in vacuo. The solid γ-spirolactams **1** crystallized from *n*-hexane in analytically pure form and afforded single crystals for X-ray measurements.
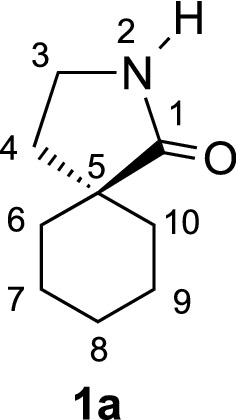


#### γ-Spirolactam **1a**

White solid (3.00 g, 98%). *R*_f_ = 0.21 (EtOAc); m.p. 107–108 °C; ^1^H NMR (500 MHz, CDCl_3_): *δ* 7.45 (brs, 1 H, N–H), 3.24 (t, *J *= 7.0 Hz, 2 H, 3-H), 1.94 (t, *J *= 7.0 Hz, 2 H, 4-H), 1.18–1.70 (m, 10 H, 6-H–10-H); ^13^C NMR (125 MHz, CDCl_3_): *δ* 22.4 (t, C-7, C-9), 25.6 (t, C-8), 31.7 (t, C-4), 32.2 (t, C-6, C-10), 39.2 (t, C-3), 44.2 (s, C-5), 183.7 (s, C-1); IR (KBr) $\widetilde{v}$ = 3292, 2928, 1686, 1650, 1279, 1071, 739 cm^−1^; elemental analysis calcd (%) for C_9_H_15_NO (153.22): C 70.55, H 9.87, N 9.14; found: C 70.29, H 9.99, N 9.07.
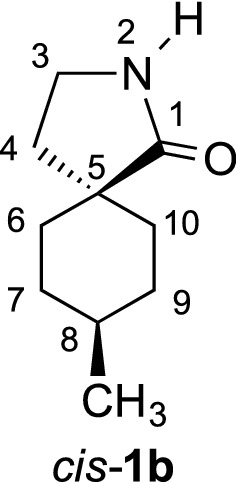


#### γ-Spirolactam *cis*-**1b**

White solid (3.20 g, 96%). *R*_f_ = 0.26 (EtOAc); m.p. 128–129 °C; ^1^H NMR (600 MHz, CDCl_3_): *δ* 7.69 (brs, 1 H, N–H), 3.22 (t, *J *= 7.0 Hz, 2 H, 3-H), 1.89 (t, *J *= 7.0 Hz, 2 H, 4-H), 1.54–1.40 (m, 4 H, 7-H_ax._, 9-H_ax._, 6-H_equ._, 10-H_equ._), 1.37–1.41 (m, 2 H, 7-H_equ._, 9-H_equ._), 1.27–1.34 (m, 1 H, 8-H), 0.89 (ddd, *J *= 15.7, 9.8, 4.7 Hz, 2 H, 6-H_ax._, 10-H_ax._), 0.81 (d, *J *= 6.7 Hz, 3 H, CH_3_); ^13^C NMR (75 MHz, CDCl_3_): *δ* 22.6 (q, CH_3_), 31.1 (t, C-7, C-9), 31.3 (t, C-4), 31.9 (d, C-8), 32.1 (t, C-6, C-10), 39.2 (t, C-3), 44.0 (s, C-5), 184.0 (s, C-1); IR (KBr) $\widetilde{v}$ = 3201, 2918, 1680, 1287, 753 cm^−1^; elemental analysis calcd (%) for C_10_H_17_NO (167.25): C 71.81, H 10.24, N 8.38; found: C 71.60, H 10.31, N 8.44.
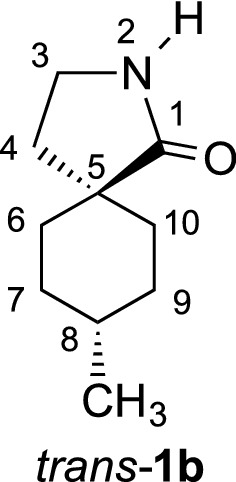


#### γ-Spirolactam *trans*-**1b**

White solid (3.24 g, 97%). *R*_f_ = 0.35 (EtOAc); m.p. 114–115 °C; ^1^H NMR (600 MHz, CDCl_3_): *δ* 6.70 (brs, 1 H, N–H), 3.26 (t, *J *= 6.9 Hz, 2 H, 3-H), 1.89 (t, *J *= 6.9 Hz, 2 H, 4-H), 1.85 (ddd, *J *= 13.3, 8.1, 3.0 Hz, 2 H, 6-H_equ._, 10-H_equ._), 1.48–1.57 (m, 5 H, 7-H, 8.H, 9-H), 1.24 (ddd, *J *= 13.3, 8.4, 4.1 Hz, 2 H, 6-H_ax._, 10-H_ax._), 0.96 (d, *J *= 6.2 Hz, 3 H, CH_3_); ^13^C NMR (75 MHz, CDCl_3_): *δ* 20.0 (q, CH_3_), 29.3 (t, C-7, C-9), 29.8 (d, C-8), 31.2 (t, C-6, C-10), 35.3 (t, C-4), 38.8 (t, C-3), 42.2 (s, C-5), 183.0 (s, C-1); IR (KBr) $\widetilde{v}$ = 3211, 2924, 1677, 1289, 755 cm^−1^; elemental analysis calcd (%) for C_10_H_17_NO (167.25): C 71.81, H 10.24, N 8.38; found: C 71.71, H 10.12, N 8.37.

#### X-ray structure analysis

The crystals were embedded in perfluoropolyalkylether oil and mounted within a MicroGripper. The data collections were performed at 210 K on a STOE StadiVari diffractometer equipped with a four-circle goniometer (open Eulerian cradle), a Genix Microfocus X-ray source (Mo) with a graded multilayer mirror and a Dectris 200 K detector (Δ*ω *= 0.5°; detector distance 60 mm; **1a**: 4110 frames, 5 s exposure time per frame; ***cis*-1b**: 2488 frames, 10 s exposure time per frame; ***trans*-1b**: 2516 frames, 10 s exposure time per frame). The data were corrected for absorption as well as for Lorentz and polarization effects using the program X-Area [16]. The structures were solved by direct methods using SHELXS-2013/2  [17] and refined by full-matrix least squares on *F*^2^ using the program SHELXL-2014/7 [18]. All non-hydrogen atoms were refined anisotropically. The hydrogen atoms of the N–H groups were located from the difference Fourier maps and free refined. The other hydrogen atoms were calculated in their expected positions using a riding model with C–H = 0.98 Å (–CH_2_) and C–H = 0.97 Å (–CH_3_), allowing for rotation), and *U*_iso_(H) = 1.2*U*_eq_(CH_2_) and *U*_iso_(H) = 1.5*U*_eq_(CH_3_). For the visualization the programs ORTEP-3 for windows [19] and DIAMOND [20] were used.

### Crystal data of 1a

C_9_H_15_NO, *M* = 153.22, monoclinic, *a* = 20.4582(6), *b* = 11.8454(4), *c* = 28.4142(10) Å, *β* = 106.017(5)°, *V* = 6815.2(4) Å^3,^ *T* = 210(2) K, space group *C*2/*c* (no. 15), Z = 32, *μ*(Mo*K*α) = 0.077 mm^−1^; 92,264 reflections measured, 5983 unique (*R*_int_ = 0.0616) which were used in all calculations. Final *R* values: *wR*_2_(*F*^2^) = 0.1079, *R*_1_ = 0.0697 (all data); *wR*_2_(*F*^2^) = 0.0969, *R*_1_ = 0.0373 [*I* > 2*σ*(*I*)].

### Crystal data of *cis*-1b

C_10_H_17_NO, *M* = 167.24, monoclinic, *a* = 14.7946(9), *b* = 6.4589(2), *c* = 10.5769(7) Å, *β* = 105.199(5)°, *V* = 975.34(10) Å^3^, *T* = 210(2) K, space group *P*2_1_/*c* (no. 14), Z = 4, *μ*(Mo*K*α) = 0.073 mm^−1^; 15,495 reflections measured, 1713 unique (*R*_int_ = 0.0236) which were used in all calculations. Final *R* values: *wR*_2_(*F*^2^) = 0.0940, *R*_1_ = 0.0391 (all data); *wR*_2_(*F*^2^) = 0.0913, *R*_1_ = 0.0336 [*I* > 2*σ*(*I*)].

### Crystal data of *trans*-1b

C_10_H_17_N O, *M* = 167.24, monoclinic, *a* = 11.1386(5), *b* = 10.5587(5), *c* = 8.3892(7) Å, *V* = 948.34(10) Å^3^, *T* = 210(2) K, space group *P*2_1_/*c* (no. 14), Z = 4, *μ*(Mo*K*α) = 0.073 mm^−1^; 14,814 reflections measured, 1655 unique (*R*_int_ = 0.0261) which were used in all calculations. Final *R* values: *wR*_2_(*F*^2^) = 0.0847, *R*_1_ = 0.0368 (all data); *wR*_2_(*F*^2^) = 0.0816, *R*_1_ = 0.0313 [*I* > 2*σ*(*I*)].

## Additional files

**Additional file 1.** Crystallographic information file of **1a.**

**Additional file 2.** Crystallographic information file of cis-**1b.**

**Additional file 3.** Crystallographic information file of trans-**1b.**

## Data Availability

Additional files 1, 2, and 3 include crystallographic information files (CIF). CCDC 1812882 (**Ia**), CCDC 1812884 (*cis*-**1b**), and CCDC 1812883 (*trans*-**1b**) contain the supplementary crystallographic data for this paper. These data can be obtained free of charge from the Cambridge Crystallographic Data Centre via http://www.ccdc.cam.ac.uk/data_request/cif.

## Abbreviations

NMR: nuclear magnetic resonance
NOE NMR: nuclear overhauser effect NMR
2D: 2-dimensional
FT-IR: Fourier transformation IR
m.p.: melting point
TLC: thin layer chromatography
COSY: correlated spectroscopy
HMQC: heteronuclear multiple quantum coherence
HMBC: heteronuclear multiple bond correlation
IR: infrared
